# Exploring the Role of TSPO-PET Imaging Among MRI-Negative Patients with Temporal Lobe Epilepsy: From the Perspective of Heterogeneity

**DOI:** 10.3390/brainsci16020246

**Published:** 2026-02-22

**Authors:** Yuncan Chen, Jing Wang, Shimin Xu, Qinyue Wang, Shuhao Mei, Jiaying Lu, Yiqiao Wang, Huamei Lin, Dongyan Wu, Liang Chen, Chuantao Zuo, Yihui Guan, Jingjie Ge, Xunyi Wu

**Affiliations:** 1 Department of Neurology, Huashan Hospital, Fudan University, Shanghai 200040, China; 22111220035@m.fudan.edu.cn (Y.C.);; 2 Department of Nuclear Medicine, Huashan Hospital, Fudan University, 518 East Wuzhong Road, Shanghai 200235, Chinalovejingjie@fudan.edu.cn (J.G.); 3 PET Center, Huashan Hospital, Fudan University, 518 East Wuzhong Road, Shanghai 200235, China; 4 Department of Neurosurgery, Huashan Hospital, Fudan University, Shanghai 200032, China; 5 State Key Laboratory of Medical Neurobiology, Fudan University, Shanghai 200032, China; 6 MOE Frontiers Center for Brain Science, Fudan University, Shanghai 200032, China; 7 Institutes of Brain Science, Fudan University, Shanghai 200032, China; 8 National Center for Neurological Disorders, 12 Wulumuqi Zhong Road, Shanghai 200040, China

**Keywords:** temporal lobe epilepsy, neuroinflammation, TSPO, asymmetry indices, heterogeneity

## Abstract

**Highlights:**

**What are the main findings?**

- TSPO-PET/MRI with [18F] DPA-714 revealed marked inter-individual heterogeneity in neuroinflammatory distribution among temporal lobe epilepsy patients.
- Elevated TSPO uptake and asymmetry index were significantly associated with seizure frequency and the interval between the last seizure and PET imaging.

**What are the implications of the main findings?**

- TSPO-PET may serve as a sensitive tool for localizing epileptogenic foci, particularly in MRI-negative temporal lobe epilepsy.
- Clinical seizure-related factors like scanning timing should be considered when interpreting TSPO-PET signals, as they may contribute to variability in neuroinflammatory imaging patterns.

**Abstract:**

**Background/Objectives**: This study explored the heterogeneous distribution pattern of translocator protein 18kDa (TSPO)-PET/MRI using radioligand [^18^F] DPA-714 in temporal lobe epilepsy patients and identified clinical factors influencing imaging outcomes. **Methods**: The TSPO imaging in individual patient was evaluated with both visual reading and quantitative assessment using an asymmetry index based on cerebellum-normalized standardized uptake values. The association between clinical factors and TSPO imaging outcomes was assessed. Pathological evaluation was conducted in three patients. **Results**: Twenty-nine TLE patients and ten healthy controls were enrolled. Visual evaluation revealed increased [^18^F] DPA-714 uptake in twenty patients as compared to controls, predominantly in a unilateral regional brain, while the remaining nine patients showed visually undetectable uptake of [^18^F] DPA-714. Consistently, quantitative analysis revealed that 69% (20/29) patients exhibited at least one brain area with significant asymmetry index, notably in the temporal lobe (85%, 17/20). A high asymmetry index could also be observed in the parietal (13.8%, 4/29) and occipital lobe (17.2%, 5/29). Significant associations were identified between the asymmetry index and seizure frequency (*p* = 0.045, OR = 7.994), and the interval from last seizure to PET scan (*p* = 0.033, OR = 6.712). Moreover, we confirmed the pathology in three patients via immunohistochemistry, which underscored the potential of TSPO-PET in detecting minor lesion. **Conclusions**: TSPO-PET reveals patient-specific and network-level neuroinflammatory heterogeneity in MRI-negative TLE, supporting its potential role as a complementary tool for presurgical evaluation.

## 1. Introduction

Epilepsy stands as one of the most prevalent chronic neurological conditions, marked by recurrent unprovoked seizures, impacting over 70 million individuals globally [1]. Despite the availability of more than 20 anti-seizure medications in clinical practice, it is estimated that epilepsy contributes to 0.5% of the total disease burden [2]. Among those affected, approximately one-third develop drug-resistant epilepsy, a figure that escalates to 50–70% in patients with temporal lobe epilepsy (TLE), the most common form of focal onset epilepsy [3,4]. Typical manifestations of TLE include a fixed stare, impaired awareness, confusion, and automatisms such as finger fumbling or lip-smacking [5]. Given the heightened risk of drug resistance and disease progression, many TLE patients seek neurosurgical interventions during the middle or later stages of their condition, which currently represent the sole potentially curative approach. Notably, a randomized trial spanning two decades demonstrated the superiority of surgery over prolonged medical therapy in TLE patients [6]. However, the precise localization of the epileptic zone, a pivotal determinant of surgical outcomes, remains a formidable challenge [7].

At present, magnetic resonance imaging (MRI), fluorodeoxyglucose-positron emission tomography (FDG-PET), and video-electroencephalography (VEEG) are the foremost established and accessible imaging modalities, offering comprehensive presurgical structural and functional insights. FDG-PET operates by measuring cerebral glucose metabolism using a radiolabeled glucose analog to gauge neuronal activity [8]. Leveraging this principle, neuronal hypometabolism within epileptic foci during the interictal period has been well-documented [9,10]. The advent of FDG-PET has significantly advanced the localization of epileptic zones, particularly in cases where MRI or VEEG yield inconclusive results. However, interictal FDG-PET struggles to precisely delineate the surgical margin, as hypometabolic regions often extend beyond the epileptogenic zone [9]. In a study, FDG-PET identified 25 cases of the same region as the intracranial EEG monitoring identified among 45 cases (56% sensitivity) [11]. Consequently, there is a pressing clinical need to explore novel radiotracers with enhanced sensitivity and specificity to achieve accurate localization of the epileptic zone.

Emerging evidence underscores the activation of neuroinflammation in epileptogenic brain regions, both in patient specimens and animal models [12], this activation is characterized by reactive microglia and subsequently activated astrocytes, alongside the upregulation of inflammatory cytokines [13,14]. Given its involvement in epilepsy initiation and progression, neuroinflammation holds promise as a potential biomarker for neuroimaging-based epileptic zone localization. The translocator protein 18 kDa (TSPO) has long been investigated as a biomarker of reactive gliosis and inflammation associated with various neuropathological conditions [15]. In epilepsy, TSPO has been shown to be overexpressed in activated microglia [16]. Consequently, research endeavors are emerging to assess the utility of TSPO ligands as radiotracers for identifying lesions in epilepsy patients. Hirvonen et al. observed an increased uptake of radioactivity ipsilateral to the seizure focus in patients with TLE (n = 16), particularly in the hippocampus and amygdala, following injection of [^11^C] PBR-28, indicative of heightened TSPO expression [17]. Moreover, this asymmetry was more pronounced in patients with hippocampal sclerosis. In a study with a larger sample size (n = 23), Gershen et al. further explored this asymmetry by comparing two second-generation ligands, [^11^C] PBR-28 and [^11^C] DPA-713 [18]. They noted that relative [^11^C] PBR-28 and [^11^C] DPA-713 uptakes were higher ipsilateral than contralateral to seizure foci in patients with TLE, with the latter demonstrating more significant asymmetry. Similar investigations have been conducted in populations with neocortical epilepsy [19] and pediatric cohorts [20]. In a recent study, researchers proposed that an inter-hemispheric asymmetry index in the hippocampus, measured using TSPO-PET imaging in patients with TLE, exhibited excellent test–retest reliability. This suggests that this biomarker could serve as a method of evaluation in clinical trials for epilepsy [21].

However, limited attention has been given to some critical questions: Does TSPO-PET consistently demonstrate regionally elevated uptake in all patients with TLE? If not, what clinical factors modulate this variability? Furthermore, how effective is TSPO-PET in patients with negative MRI findings? Answers to these inquiries could not only guide clinicians in recommending TSPO-PET to patients but also enhance our understanding of the mechanisms underlying epileptogenesis, particularly regarding neuroinflammation. In this study, we sought to validate previous observations of heightened TSPO ligand uptake ipsilateral to epileptic foci in TLE patients, utilizing a novel second-generation ligand, [^18^F] DPA-714, and incorporating more rigorous brain areas into analysis. Additionally, we aimed to explore the heterogeneity of TSPO-PET/MRI findings and identify potential factors contributing to divergent outcomes of this imaging modality in TLE patients.

## 2. Materials and Methods

### 2.1. Participants and Inclusion Criteria

This observational prospective study protocol was conducted in accordance with the principles of the Declaration of Helsinki and approved by the Ethics Committee of the Huashan Hospital (Ethics approval number: KY2023-470). Written informed consent was obtained from all individual participants in the study. Twenty-nine patients diagnosed with definite TLE from January 2024 to November 2024 at the Department of Neurology, Huashan Hospital were enrolled in our study. All clinical files and instrumental records of patients underwent a thorough review by at least two independent clinicians. Diagnosis was established based on clinical manifestations, MRI findings, and VEEG results following the guidelines set forth by the International League Against Epilepsy [22]. Briefly, TLE is typically characterized by a blank stare accompanied by impaired awareness and seizures automatism [23]. Patients may exhibit unawareness or confusion regarding their surroundings, along with finger fumbling or lip-smacking movements, lasting from 30 s to several minutes, occasionally progressing to generalized tonic–clonic jerking. The most common auras are feelings of déjà-vu or some stomach upset. Inclusion criteria comprised a diagnosis of TLE, as well as good compliance. Detailed documentation of the following clinical characteristics was performed: sex, age, family history of epilepsy, onset of seizures, current seizure frequency, interval from the last seizure to the scan, and current anti-seizure medications regimen. Ten age-matched healthy controls (5 females and 5 males; mean age 30 years) were enrolled to exclude possible physiological left-right TSPO asymmetry.

### 2.2. TSPO PET/MRI Protocol

A uPMR790 HD TOF PET/MR instrument (United Imaging Healthcare, Shanghai, China), composed of a 3.0-Tesla MR imager and a fully integrated PET detector, was used for the cerebral PET/MR acquisition. For [^18^F] DPA-714 PET/MRI, a mean dose of 370 MBq of [^18^F] DPA-714 was injected intravenously, and the static PET acquisition was measured 40 min after injection and lasted 50 min in individual patients [24,25]. PET images were co-registered to simultaneously acquired, routine non-contrast enhanced MR images (T1, T2, T2 FLAIR). All scans were independently reviewed by two experienced epilepsy neuroradiologists, and patients were classified as MRI-negative only when no structural epileptogenic lesions were identified by consensus. All participants remained awake during the course of the uptake and scanning procedure. PET acquisitions were performed without antiseizure medication withdrawal.

### 2.3. Visual Reading and Quantitative Analysis of TSPO Imaging

Three imaging radiologists, blinded to patients’ clinical information, conducted a visual assessment of the TSPO-PET/MRI scan to determine whether there was high uptake in the frontal, temporal and insular, parietal, and occipital lobes, as well as the cerebellum. If there was dissent, the majority opinion was followed. Scans were classified as visually positive if a clear focal or lateralized increase in TSPO uptake was observed, and as visually negative or undetectable if no such abnormality was identified. Discrepancies were resolved by consensus. As for quantitative analysis, the segmentation of regions of interest was performed using the PET/MRI of individual patients with TLE in combination with automated anatomical labeling atlas with 27 brain regions, which were assigned to the following anatomical compartments: bilateral frontal cortex, parietal cortex, temporal cortex (including limbic system), occipital cortex and cerebellum hemisphere. Anatomical regions were defined using the AAL atlas implemented in PMOD (PMOD Technologies LLC, Zurich, Switzerland), with automated segmentation followed by visual quality control. The mean standardized uptake values (SUVs) of [^18^F] DPA-714 in the global region from TLE patients were extracted. The obtained SUVs in each region were subsequently divided by the average SUV of bilateral cerebellum, namely cerebellum normalized SUVs ratio (SUVRc) [26]. Partial volume correction was not applied in this study.

The regional asymmetry of TSPO was calculated using the formula: [(ipsilateral − contralateral)/(ipsilateral + contralateral)] × 200%, based on the SUVRc for all pairs of homologous regions of the hemispheres [18,19]. The ipsilateral hemisphere referred to the hemisphere where the clinically defined epileptic foci were located, while the contralateral hemisphere referred to the opposite hemisphere. The hemisphere where clinically defined epileptic foci are located were determined based on the patient’s clinical manifestations and VEEG results. The asymmetry index was utilized to identify focal brain regions with increased neuroinflammation and to quantify its severity. PET positivity was defined as the presence of visually or quantitatively increased TSPO uptake exceeding the predefined asymmetry threshold. The asymmetry index cutoff was defined a priori as 15%, based on the distribution of asymmetry indices in healthy controls (mean + 2 SD = 14.2%).

### 2.4. DNA Extraction and Single Nucleotide Polymorphism Genotyping

In consideration of the binding ability of radioligand ([^18^F] DPA-714) to TSPO determined by genetic polymorphism of the TSPO gene, all patients that underwent PET scans received blood tests for genotyping. Genomic DNA was extracted from whole blood with DNA kit (QIAGEN GmbH, Hilden, Germany) following the manufacturer’s protocol. DNA quality was assessed by optical absorbance. Exon 4 of the TSPO gene containing the polymorphism rs6971 (Ala or Thr at position 147) was polymerase chain reaction amplified. The polymerase chain reaction product was tested with gel electrophoresis and sequenced using the Sanger method. Genotypes of rs6971 encoding amino acids were categorized as high-affinity binders (HABs; C/C; Ala/Ala), medium-affinity binders (MABs; C/T; Ala/Thr), and low-affinity binders (LABs; T/T; Thr/Thr) [27].

### 2.5. Stereo-EEG Electrode Implantation and Recording

Preoperative planning combined MRI, PET and scalp EEG data, all integrated within a specialized neuronavigational platform to determine electrode targets. Under general anesthesia, depth electrodes were stereotactically placed along orthogonal or oblique paths. Correct positioning was then verified by postoperative MRI. Intracranial EEG was continuously recorded over several days in a monitored inpatient setting, capturing both interictal and ictal events via a clinical video-EEG system. Experienced epileptologists subsequently reviewed the recording—focusing on interictal epileptiform discharges and seizure-onset pattern—to localize aberrant activity in concordance with the imaging findings.

### 2.6. Immunohistochemistry

Resected brain tissues were fixed in 10% buffered formalin, embedded in paraffin, and sectioned at a thickness of 5 μm. Hematoxylin and eosin staining was performed to evaluate general cytoarchitecture and identify structural abnormalities such as dysmorphic neurons or balloon cells. Immunohistochemical staining was conducted using established histopathological protocols with specific antibodies targeting ionized calcium-binding adaptor molecule 1 (IBA-1) for microglia (catalog no. ab178846, Abcam, Cambridge, UK), glial fibrillary acidic protein (GFAP) for astrocytes (catalog no. ab68428, Abcam, Cambridge, UK), neuron-specific nuclear binding protein (NeuN) for neurons (catalog no. 26975-1-AP, Proteintech, Rosemont, IL, USA), and SMI-32 for non-phosphorylated neurofilament-H (catalog no. 801702, BioLegend, San Diego, CA, USA).

### 2.7. Statistical Analysis

Continuous variables were presented as means ± standard deviation for normally distributed data, while the median and inter-quartile range (IQR) were used for non-normally distributed data. Categorical variables were reported as counts and percentages. To examine the correlation between TSPO-PET/MRI outcomes and relevant clinical factors, logistic regression analysis was conducted. The significance level was set at a 95% confidence limit, with a two-tailed *p*-value < 0.05 considered statistically significant. We performed statistical analyses using Graphpad Prism (Version 7.00.).

## 3. Results

### 3.1. Clinical, MRI and VEEG Characteristics of Study Population

**Figure 1:**
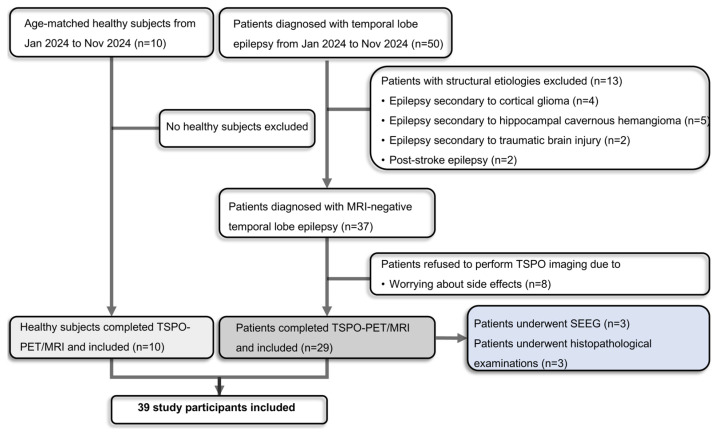
Flow diagram of the study participants recruitment. TSPO-PET, translocator protein 18 kDa-positron emission tomography.

A flow diagram of the study participants is shown in Figure 1. Patients with structural etiologies such as cortical glioma, hippocampal cavernous hemangioma, traumatic brain injury and stroke were excluded. Finally, twenty-nine patients diagnosed as MRI-negative TLE (15 female and 14 male patients) were continuously enrolled in this study as shown in Table S1. The patients’ median age of seizure onset was 24 years (IQR: 13.5–35.5 years). The median interval from seizure-onset to PET scan was seven years (IQR: 1–9 years), and the median interval from last seizure to PET scan was nine days (IQR: 5.5–14 days). Among the patients, nearly half (13/29) presented with focal impaired awareness seizures, featured by impaired awareness with automatism. Additionally, some patients (5/29) were classified as having focal to bilateral tonic–clonic seizures due to the development of bilateral tonic–clonic seizure activity as observed in video records or described by witnesses. Notably, eleven patients (11/29) exhibited both seizure types throughout their disease course. 24 of 29 patients demonstrated hypometabolism on FDG-PET. As for the rs6971 polymorphism, 24 patients were classified as HABs, and five were MABs, no LABs were identified.

In terms of VEEG findings, all patients, except for Case 4 and Case 12, demonstrated epileptic discharge characterized by sharp waves, spikes, and sharp and slow waves complex. Among these patients, sixteen presented with epileptic discharge in their left hemisphere, while abnormal discharge was detected in the right hemisphere in eleven patients. Furthermore, though two patients had a normal VEEG recording, their seizure symptoms were characterized by right limbs automatism accompanied by impaired awareness. Notably, among patients with laterally abnormal discharge, the majority of patients displayed restricted discharge primarily in the temporal lobe, similar to Case 5 (Figure S1a). However, a few patients exhibited diffused discharge dominant in the temporal lobe but spreading to other regions of the ipsilateral hemisphere, such as Case 9 (Figure S1b) and Case 10 (Figure S1c). Regarding MRI, all the patients included in this study had normal MRI results.

### 3.2. Visual and Quantitative Assessment of [^18^F] DPA-714 Imaging

**Figure 2:**
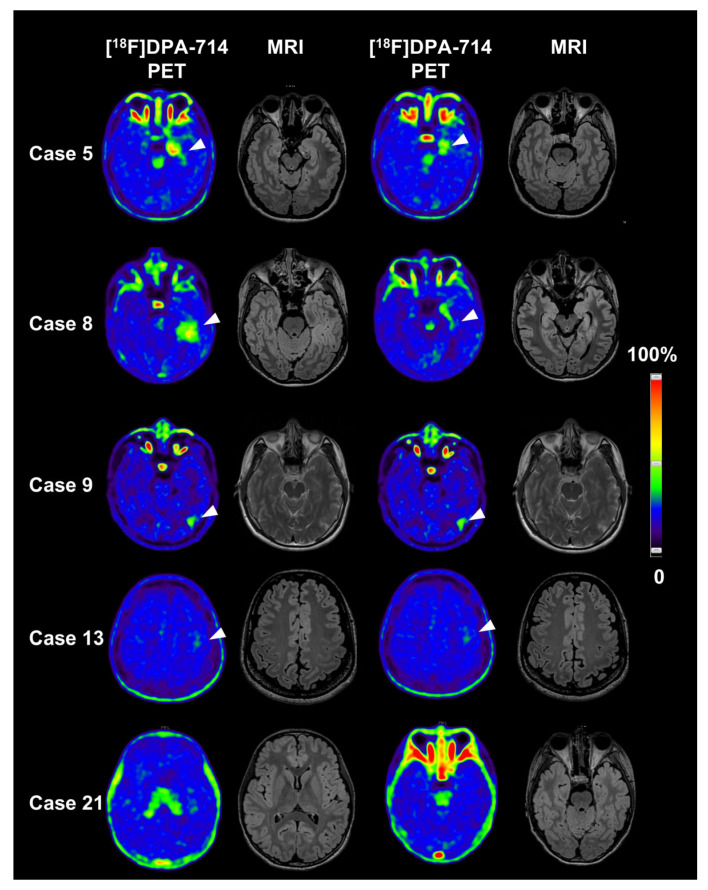
Representative TSPO-PET/MRI pattern in patients with TLE. Case 5 and Case 8 presented focal high [^18^F] DPA-714 uptake in left temporal lobe (white arrowhead) while Case 9 exhibited focal high uptake in left occipital lobe (white arrowhead). Case 13 showed a regional higher signal in left frontal lobe. Case 21 displayed very low or visually undetectable uptake. TSPO, translocator protein 18 kDa; PET, positron emission tomography; MRI, magnetic resonance imaging; TLE, temporal lobe epilepsy.

The results of the visual analysis of TSPO imaging are summarized in Table S1. Twenty out of twenty-nine patients exhibited increased uptake of [^18^F] DPA-714, with 55.2% (16/29) involving the temporal lobe, 13.8% (4/29) involving the occipital lobe, 13.8% (4/29) involving the parietal lobe and 3.4% (1/29) involving the frontal lobe. Notably, the hemisphere where the potential epileptic foci were located, as clinically defined by VEEG and symptoms, was completely consistent with the findings of visual evaluation of TSPO imaging scans among those observed with abnormalities. As depicted in Figure 2—exemplary images of five patients—many displayed abnormally increased regional retention restricted to the temporal lobe, as seen in Case 5, and Case 8. Interestingly, Case 9 exhibited a limited lesion in occipital lobe, despite typical symptoms of TLE and significant discharge originating from the temporal lobe on VEEG, suggesting potential inflammation spread and interlobe communication between the temporal and occipital lobes [28]. Additionally, Case 13 showed a regional slightly high signal in the left frontal lobe. Furthermore, a minority of patients (31%, 9/29) displayed very low and visually undetectable uptake of [^18^F] DPA-714, akin to Case 21, after excluding physiological uptake in the choroid plexus.

**Figure 3:**
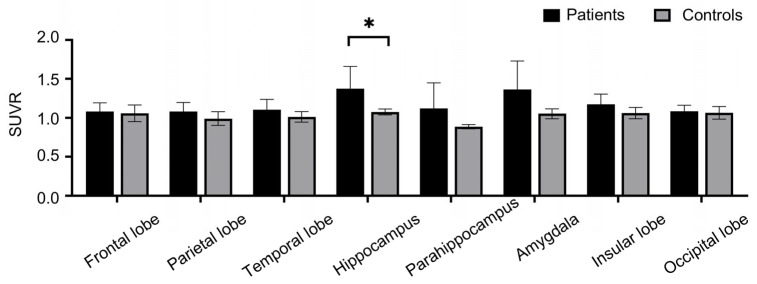
The group comparison of [^18^F] DPA-714 uptake among patients vs. controls in main brain regions. TLE patient group demonstrated significantly elevated uptake of [^18^F] DPA-714 in the hippocampus (*p* = 0.028). The trend of increased uptake could also be seen in the parahippocampus and amygdala in TLE patient group, though it did not reach statistical significance (*p* = 0.13 and *p* = 0.069, respectively). We did not observe significant group difference in the whole temporal, frontal, parietal and occipital cortices. * *p* < 0.05. TLE, temporal lobe epilepsy.

The uptake of [^18^F] DPA-714 in the hemisphere where the clinically defined epileptic foci were located, was illustrated in the form of a heat map (Figure S2). In the group comparison with healthy controls, the TLE patient group had significantly elevated uptake of [^18^F] DPA-714 in the hippocampus (*p* = 0.028, Figure 3), which was consistent with the previous study [18]. The trend of increased uptake could also be seen in the parahippocampus and amygdala in TLE patient group, though it did not reach statistical significance (*p* = 0.13 and *p* = 0.069, respectively). After false discovery rate (FDR) correction, only the hippocampal region showed a significant difference between groups (*p* = 0.001, q = 0.007). There was no obvious difference between the two groups in the whole temporal, frontal, parietal and occipital cortices.

### 3.3. Quantitative Analysis of Asymmetric [^18^F] DPA-714 Uptake

**Figure 4:**
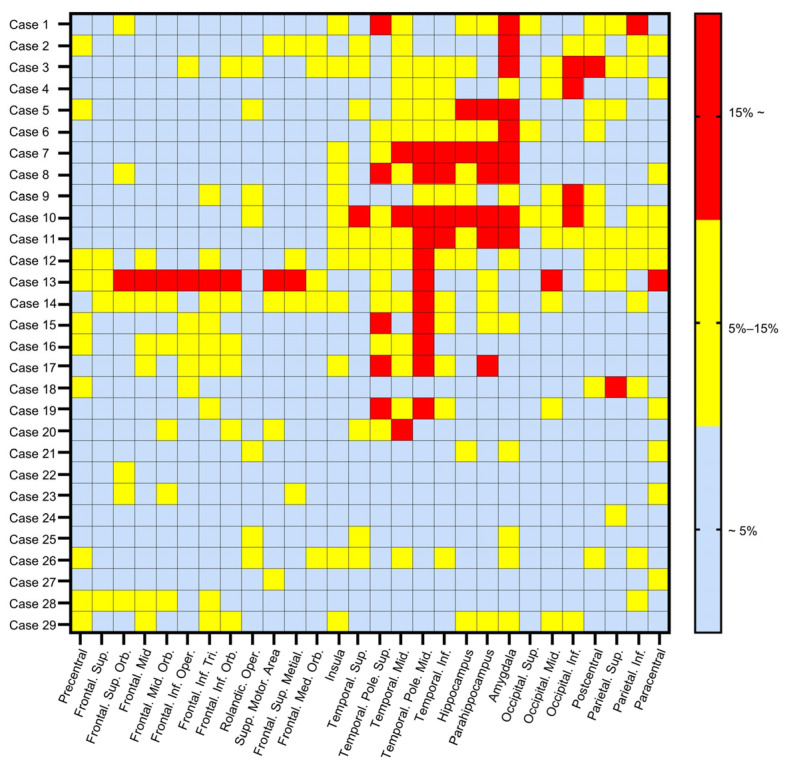
Heat map of asymmetry index of [^18^F] DPA-714 uptake among patients with TLE. 20 patients had at least one brain area with an asymmetry index higher than 15%. These areas were predominantly located in the temporal lobe (85%, 17/20). TLE, temporal lobe epilepsy.

To further visualize the heterogeneous uptake pattern, we presented the asymmetry index of each brain area among individual patients (Figure 4). Our analysis revealed that among the patients observed with abnormalities with visual reading, all had at least one brain area with an asymmetry index higher than 15%. These areas were predominantly located in the temporal lobe (85%, 17/20). More specifically, 35.3% (6/17) regions with high uptake were situated in the hippocampus/parahippocampus, and 52.9% (9/17) in the amygdala. At times, the brain area with the highest asymmetry index was located outside the temporal lobe, such as in the parietal lobe (13.8%, 4/29) and occipital lobe (17.2%, 5/29). In addition, the remaining nine patients who showed visually undetectable uptake of [^18^F] DPA-714 exhibited a relatively low degree of asymmetry index across brain areas (less than 15%). The details of maximum asymmetry index in individual patients with TLE (anatomical region, comparison with ten age-matched controls in quantification, the mean 95% confidence intervals) were illustrated in the Table S2.

### 3.4. Clinical Factors Related to Heterogeneity

**Figure 5:**
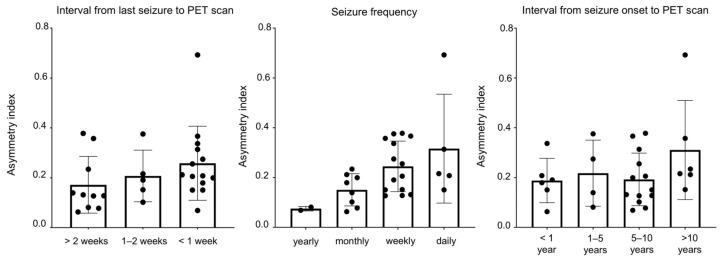
Asymmetry index of [^18^F] DPA-714 uptake and associated factors among patients with TLE. There is a positive correlation between the PET outcome and seizure frequency, interval from seizure onset to PET scan. PET, positron emission tomography; TLE, temporal lobe epilepsy.

We meticulously investigated these clinical factors, particularly seizure frequency, interval from seizure onset to PET scan, and interval from the last seizure to PET scan, as delineated in Table S1. Through logistic regression analysis, we discerned significant associations between the PET outcome and the interval from the last seizure to the PET scan (*p* = 0.033, OR = 6.712, 95% CI 1.163–38.726, Table 1), suggesting that a shorter interval from the last seizure to the PET scan is correlated with a higher proportion of patients with increased TSPO uptake. Concerning seizure frequency, patients with more frequent seizures tended to present a positive outcome (*p* = 0.045, OR = 7.994, 95% CI 1.05–60.842). Similarly, concerning the interval from onset to PET scan, it did not show a significant correlation between the disease course and PET result (*p* = 0.15, OR = 2.808, 95% CI 0.688–11.463). The histogram (Figure 5) provides a visual depiction of the positive correlation between the PET outcome and seizure frequency, interval from seizure onset to PET scan, as well as interval from the last seizure to PET scan. This observation further supports the notion that these clinical factors play crucial roles in influencing the outcome of PET scans in patients with TLE.

### 3.5. Heterogeneity Revealed by Electrophysiological and Histopathological Examinations

**Figure 6:**
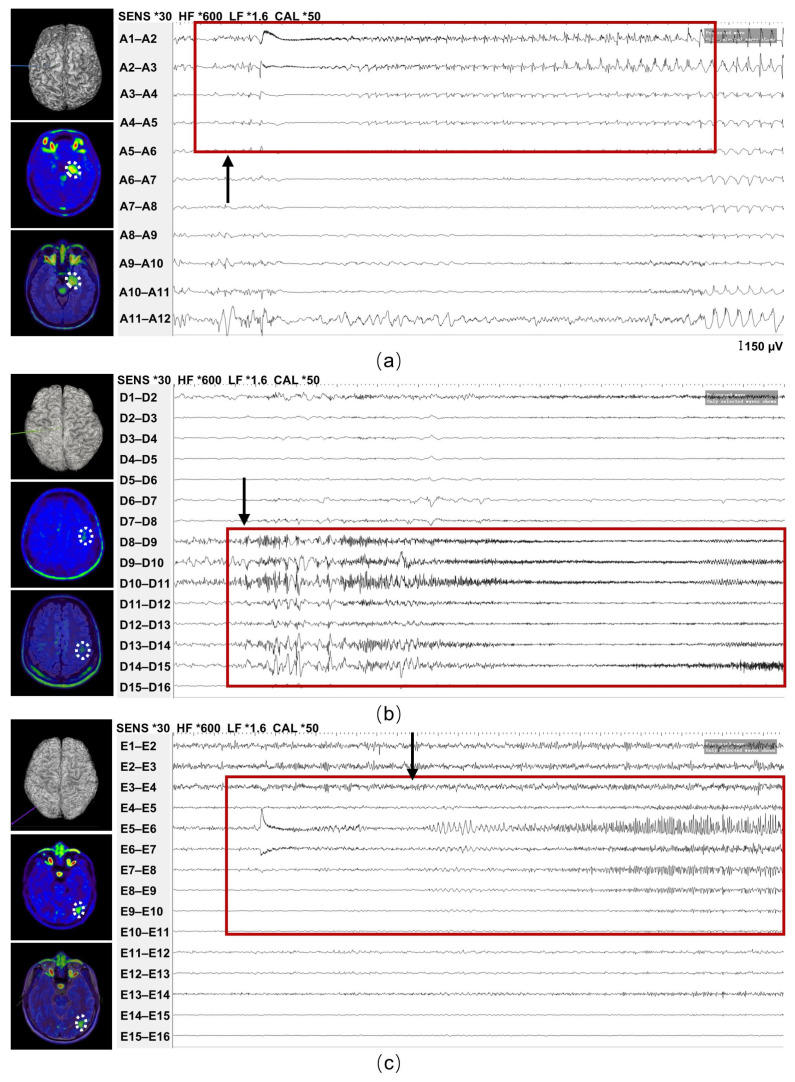
TSPO-PET imaging and SEEG findings from three representative patients demonstrating the concordance between neuroinflammation at the epileptogenic focus and abnormal epileptiform discharges. (**a**) A patient exhibited high TSPO uptake in the left mesial temporal lobe (white dotted circle), and SEEG recordings captured abnormal discharges originating from the corresponding regions. (**b**) A patient showed slightly elevated TSPO uptake in the left frontal lobe (white dotted circle), where abnormal discharges were captured. (**c**) A patient with high TSPO uptake signal in the left occipital lobe (white dotted circle), and SEEG recordings captured abnormal discharges originating from the corresponding regions. The red frames highlighted the electrode contacts showing prominent epileptiform discharges, including the seizure onset activity. The black arrow indicated the onset of the abnormal discharges. The symbol “*” indicated the applied gain or filter setting displayed by the SEEG recording system. TSPO, translocator protein 18 kDa; PET, positron emission tomography; SEEG, stereoelectroencephalography.

Interestingly, we observed that some patients demonstrated regionally elevated signals outside the temporal lobe, despite being clinically diagnosed with TLE based on their seizure semiology and VEEG. To determine whether these extra-temporal signals represented false positives or reflected underlying pathological heterogeneity, we conducted detailed electrophysiological and histopathological examinations among patients who met the clinical criteria for surgical intervention. Three patients underwent stereoelectroencephalography (SEEG) implantation to localize the epileptogenic zone. Among them, one patient exhibited high TSPO uptake in the temporal lobe, and SEEG recordings captured abnormal discharges originating from the corresponding regions (Figure 6a). Interestingly, two other patients showed elevated TSPO uptake in the frontal and occipital lobes, respectively, where SEEG also detected abnormal discharges (Figure 6b,c). These findings suggest that the TSPO hyperintensities in the frontal and occipital regions are not false-positive signals, but rather reflect potentially relevant inflammatory activity, indicating the presence of abnormal neural networks involving the temporal lobe and other brain regions in TLE patients.

**Figure 7:**
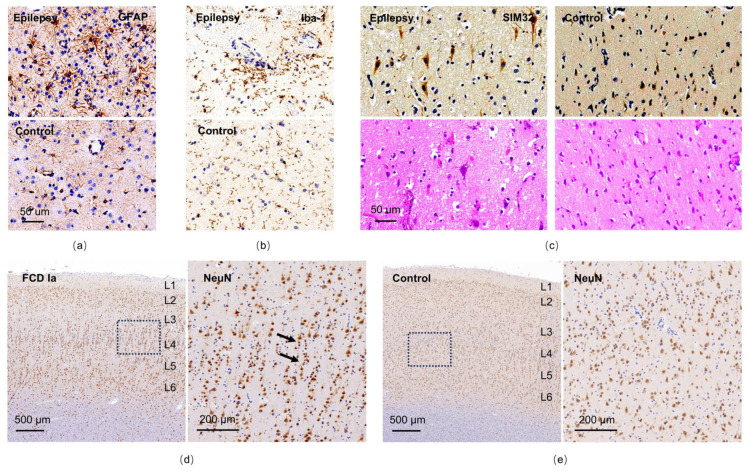
Pathological evaluation of surgical resection tissues from three patients using histological staining on paraffin-embedded sections. (**a**,**b**) GFAP and IBA-1 immunostaining reveal abnormal clustering of astrocytes and microglia within the resected tissues from one patient with TSPO hyperintensity in the occipital lobe, compared with a non-epilepsy control patient. (**c**) The patient with TSPO hyperintensity in the frontal lobe showed dysmorphic neurons with elevated expression of SMI-32. (**d**) In the cortex of a patient with TSPO hyperintensity in the temporal lobe, while the discernible six-layered architecture is preserved, small-diameter neurons forming microcolumnar arrangements (black arrow) are prominently observed by NeuN immunostaining. In contrast, the adjacent cortex exhibits normal cytoarchitecture (**e**). In (**d**,**e**), L1–L6 indicate cortical layers 1–6; the black dotted frames indicate the regions shown at higher magnification. GFAP, glial fibrillary acidic protein; IBA-1, Ionized calcium-binding adapter molecule 1; TSPO, translocator protein 18 kDa; SMI-32, non-phosphorylated neurofilament-H; NeuN, neuron-specific nuclear binding protein.

These three patients experienced recurrent seizures despite treatment with more than two anti-seizure medications, prompting their consideration for epilepsy surgery. All three subsequently underwent stereoelectroencephalography (SEEG) followed by resective surgery, with postoperative histopathological examinations conducted to confirm underlying pathology. The patient with TSPO hyperintensity in the occipital lobe showed marked aggregation of astrocytes and microglia within the resected regions (Figure 7a,b), representing typical pathological features of epileptogenic foci. In the patient with elevated TSPO uptake in the frontal lobe, immunostaining revealed dysmorphic neurons with elevated expression of SMI-32 (Figure 7c), which was the characteristics of FCD IIa. Notably, the patient with elevated signal of TSPO-PET in temporal lobe, showed a distinctive microcolumnar arrangement of small-diameter neurons in the cortex (Figure 7d), consistent with the pathological characteristics of focal cortical dysplasia (FCD) type Ia [29], while the adjacent cortical architecture appeared normal (Figure 7e). Hematoxylin-eosin staining of this patient’s cortex revealed no obvious dysmorphic neurons or balloon cells (Figure S3).

## 4. Discussion

In this study, we identified a heterogeneous distribution pattern of [^18^F] DPA-714 among patients with TLE, highlighting significant inter-individual variability in TSPO-PET imaging outcomes. Through comprehensive investigation and analysis, we confirmed the presence of associated clinical factors that interacted with the PET outcome. And notably, we demonstrated that TSPO-PET imaging held the potential to detect subtle or atypical epileptogenic lesions among MRI-negative patients like focal cortical dysplasia (FCD).

At present, resective surgery stands out as a crucial treatment option with the potential for long-term relief in patients with drug-resistant epilepsy [30]. However, during presurgical assessment, localizing the epileptogenic zone poses a significant challenge, as it is a pivotal determinant of achieving satisfactory surgical outcomes and prognosis. Among the non-invasive methods available, brain MRI remains a routine and convenient examination. Nonetheless, a notable proportion of patients, over 20% as reported [31], exhibit negative MRI findings, leading them to be considered unfavorable candidates for surgery due to the association of negative MRI results with surgical failure [32]. However, the negative finding can partially be due to the limited resolution for some small lesions like focal cortical dysplasia [33]. In our study, a majority of the enrolled patients exhibited normal brain MRI results but showed potential epileptic foci based on their reported auras, unilateral limb symptoms, and, importantly, focal discharge captured by VEEG. Consistent with previous findings, most participants displayed elevated [^18^F] DPA-714 uptake unilaterally, corresponding to their clinically defined epileptic focus, indicative of potential inflammation and microglial activation at the epileptic site. Notably, we showed that two patients with elevated signal on TSPO-PET in frontal and temporal lobe but negative MRI turned out to be FCD IIa and FCD Ia, confirmed by pathological analysis respectively, suggesting that TSPO-PET may hold promise as a precise non-invasive presurgical method.

However, from a clinical standpoint, we propose that certain variables—particularly seizure frequency and the interval between the last seizure and the PET scan—are key modulators of TSPO uptake, because not all patients with TLE exhibited high [^18^F] DPA-714 signal. Our data show that patients scanned within two weeks of a seizure were more likely to exhibit increased tracer binding, suggesting that TSPO-PET reflects seizure-induced neuroinflammation in a time-dependent manner. This is consistent with preclinical studies showing that microglial activation, the cellular basis of TSPO expression, peaks 3–14 days post-status epilepticus [14,34,35]. Thus, optimal timing of PET imaging is critical for capturing transient neuroinflammatory activity and improving diagnostic yield. What should be mentioned is, seizure timing was based on clinical history and therefore may be subject to recall bias and unrecognized subclinical seizures, which should be taken into account when interpreting temporal associations with TSPO-PET findings. In addition, seizure frequency appeared to correlate with TSPO positivity. This relationship may reflect the bidirectional interplay between seizures and inflammation: frequent seizures can exacerbate glial activation [20] and cytokine release [36,37], while sustained inflammation may perpetuate epileptogenesis through aberrant network remodeling. These findings underscore the dynamic nature of neuroinflammation in TLE and support TSPO-PET as a sensitive marker for ongoing disease activity. In this study, we took into account three important factors—seizure frequency, interval from seizure onset to PET scan, and interval from the last seizure to PET scan—using multiple logistic regression analysis, which may provide a more scientifically robust result. These factors are known to interact with inflammation activity in the epileptic focus [16,37]. Moreover, these findings have implications for clinical decision-making. Given the high costs and limited availability of TSPO-PET, identifying patients who are most likely to benefit from the scan is critical. We recommend prioritizing patients with recent seizures (within one week) and those with high seizure frequency, particularly in cases of inconclusive MRI. Such stratification enhances diagnostic efficiency, reduces unnecessary procedures, and aligns with precision medicine principles.

Neuroinflammatory responses in epilepsy are dynamic and evolve over time rather than remaining static. TSPO expression, predominantly associated with activated microglia and reactive astrocytes [38], may therefore vary across different stages of disease progression. Experimental evidence suggests that microglial activation is particularly prominent during acute and early epileptogenic phases, whereas chronic epilepsy may involve more mature gliosis or altered glial phenotypes [14]. Such stage-dependent changes in glial biology could potentially modulate TSPO expression levels and contribute to inter-individual variability in PET signal intensity. Although disease duration was not independently associated with TSPO uptake in our regression model, the temporal evolution of neuroinflammation remains a plausible biological contributor to heterogeneous imaging patterns. Longitudinal TSPO-PET studies will be necessary to clarify these dynamics.

The heterogeneity of TSPO uptake observed in this study reflects not only the influence of certain clinical factors on imaging outcomes but also the presence of extra-temporal TSPO hyperintensities, such as in the frontal and occipital lobes in some patients with TLE. It is crucial to first rule out the possibility of false-positive signals. Our SEEG recordings revealed interictal epileptiform discharges originating from these TSPO hyperintense regions in the frontal and occipital lobes. This intriguing finding preliminary indicates the nature of epilepsy as a disorder of abnormal neural networks, as well as the role of remote or secondary inflammatory responses in epileptogenesis. Potential mechanisms include glial cell-mediated propagation of neuroinflammation between brain regions and aberrant interregional neuronal projections [39], which may lead to a mismatch between the seizure onset zone and the clinically defined symptomatogenic zone. These observations warrant further investigation. Moreover, in one patient with temporal TSPO hyperintensity, histopathological analysis confirmed a diagnosis of focal cortical dysplasia (FCD) type Ia. While FCD type I is typically associated with the temporal lobe and type II with extra-temporal regions—especially the frontal lobe—our findings, supported by HE and immunohistochemical staining, revealed no balloon cells or dysmorphic neurons, but did show a microcolumnar arrangement of small neurons, consistent with previously reported pathological features of FCD type Ia [29]. Interestingly, in one patient with elevated TSPO uptake in the frontal lobe (MRI negative), histopathological analysis confirmed focal cortical dysplasia (FCD) type IIa, characterized by the presence of dysmorphic neurons with high expression of SMI-32. These two cases highlight the underlying heterogeneity among patients with a clinical phenotype of TLE, suggesting that advanced diagnostic tools such as TSPO-PET may serve as biological support in detecting epileptogenic pathology beyond conventional imaging, particular among MRI-negative patients. Among the three patients with histopathological data, two were available for postoperative outpatient follow-up. Both demonstrated a reduction in seizure frequency after surgery without new or apparent neurological deficits. However, these observations should be interpreted cautiously and require validation in larger cohorts with systematic surgical and histopathological correlation.

Several limitations in our study need to be addressed. Firstly, this study was conducted at a single center with a limited sample size. Some patients declined to undergo TSPO imaging scans due to the high cost and concerns about potential harmful effects of radiation from the tracer, leading to a limited sample size. Data from a single center may have less system error due to more consistent data collection methods, while presenting reduced generalizability. It is necessary to replicate the imaging results reported here in larger sample sizes from multi-centers, based on standardized protocols. Secondly, the AI threshold of 15% requires further validation in larger cohorts, and the limited number of healthy controls may affect statistical robustness. Thirdly, although the use of hemispheric asymmetry indices reduces dependence on the reference region and partially mitigates potential bias, the cerebellar TSPO expression may not be entirely unaffected in epilepsy which should be further investigated. Additionally, future studies incorporating partial volume correction may further refine regional quantification, particularly in cohorts with structural abnormalities. It is also necessary to incorporate measurements of arterial blood concentrations for full kinetic modeling and accurate quantification in dynamic PET, and the potential influence of ASM treatment as well as the rs6971 polymorphism on TSPO binding affinity should be considered for the absolute quantitative analysis in future studies.

## 5. Conclusions

In conclusion, our study represents the first characterization of the heterogeneous distribution pattern of TSPO-PET among patients with TLE. Through further analysis, we identified several clinical factors, notably the interval from the last seizure to the PET scan, which is significantly correlated with the outcome. Additionally, seizure frequency and the interval from onset to PET scan can also contribute to the discrepancy of TSPO-PET outcome. Importantly, the associations between TSPO uptake and clinical variables should be regarded as correlational and exploratory due to the observational design of this study. In our study, we also confirmed the pathology of three patients with focal high signal on TSPO-PET but negative MRI, via SEEG and immunostaining, which highlight the potential of TSPO-PET in detecting subtle or atypical epileptogenic lesions that escape MRI detection like FCD Ia. This study reveals the temporal dependency and spatial heterogeneity of TSPO-PET signals in temporal lobe epilepsy, offering key insights into the dynamic mechanisms and interregional propagation of neuroinflammation in epileptogenesis. However, these findings suggest that TSPO-PET reflects neuroinflammatory involvement associated with epileptic activity, rather than serving as a direct surrogate for epileptogenic pathology or a definitive presurgical localization tool. Larger, outcome-driven studies are required to establish its clinical utility.

## Figures and Tables

**Table 1:** Multiple logistic regression analysis for [^18^F] DPA-714 uptake and proportion of patients with increased TSPO uptake.

Factor	TSPO-PET/MRI Outcome	
	OR (95% CI)	*p* Value
Interval from last seizure to PET scan	6.712 (1.163–38.726)	0.033
Seizure frequency	7.994 (1.05–60.842)	0.045
Interval from seizure onset to PET scan	2.808 (0.688–11.463)	0.15

## Data Availability

The data that support the findings of this study are available from the corresponding author upon reasonable request due to privacy and ethical restrictions.

## Supplementary Materials

The following supporting information can be downloaded at: https://www.mdpi.com/article/10.3390/brainsci16020246/s1, Figure S1: Representative VEEG pattern; Figure S2: Heat map of SUVRc of [^18^F] DPA-714 uptake among patients with TLE; Figure S3: Pathological evaluation of surgical resection tissues using Hematoxylin-eosin staining; Table S1: Demographic, clinical features, VEEG monitoring, and imaging characteristics of TLE patients; Table S2: Comparison of TSPO-PET/MRI asymmetry index between TLE patients and healthy controls.

## Abbreviations

The following abbreviations are used in this manuscript:

EEG	Electroencephalography
FDG-PET	Fluorodeoxyglucose positron emission tomography
FCD	Focal cortical dysplasia
GFAP	Glial fibrillary acidic protein
HABs	High-affinity binders
IBA-1	Ionized calcium-binding adaptor molecule 1
IQR	Interquartile range
LABs	Low-affinity binders
MABs	Medium-affinity binders
MRI	Magnetic resonance imaging
NeuN	Neuron-specific nuclear binding protein
OR	Odds ratio
PET/MRI	Positron emission tomography/Magnetic resonance imaging
SEEG	Stereoelectroencephalography
SMI-32	Non-phosphorylated neurofilament-H
SNP	Single nucleotide polymorphism
SUV	Standardized uptake value
TLE	Temporal lobe epilepsy
TSPO	Translocator protein (18 kDa)
VEEG	Video-electroencephalography
