# Supplementary material for: Exploring the Role of TSPO-PET Imaging Among MRI-Negative Patients with Temporal Lobe Epilepsy: From the Perspective of Heterogeneity
Source: Brain Sci. 2026 Feb 22;16(2):246. doi: 10.3390/brainsci16020246 (PMC12938426; doi:10.3390/brainsci16020246)
Supplement: Supplementary file 1 [file brainsci-16-00246-s001.zip › brainsci-4143390-supplementary.pdf]

Supplementary material

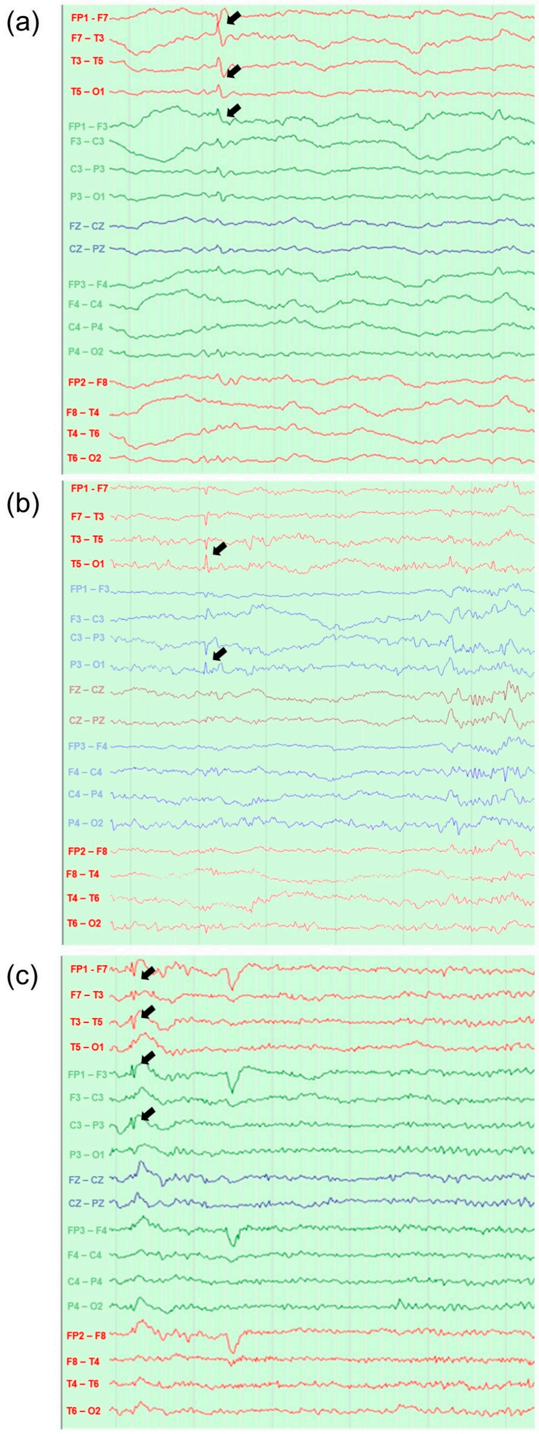

Figure S1. Representative VEEG pattern. Case 5 indicated restricted discharge in temporal lobe (a) while Case 9 presented a diffused discharge pattern arising from temporal lobe and spreading to ipsilateral parietal and occipital lobe (b). Case 10 presented a diffused discharge pattern arising from temporal lobe but spreading to ipsilateral frontal and central regions (c). VEEG, video electroencephalography

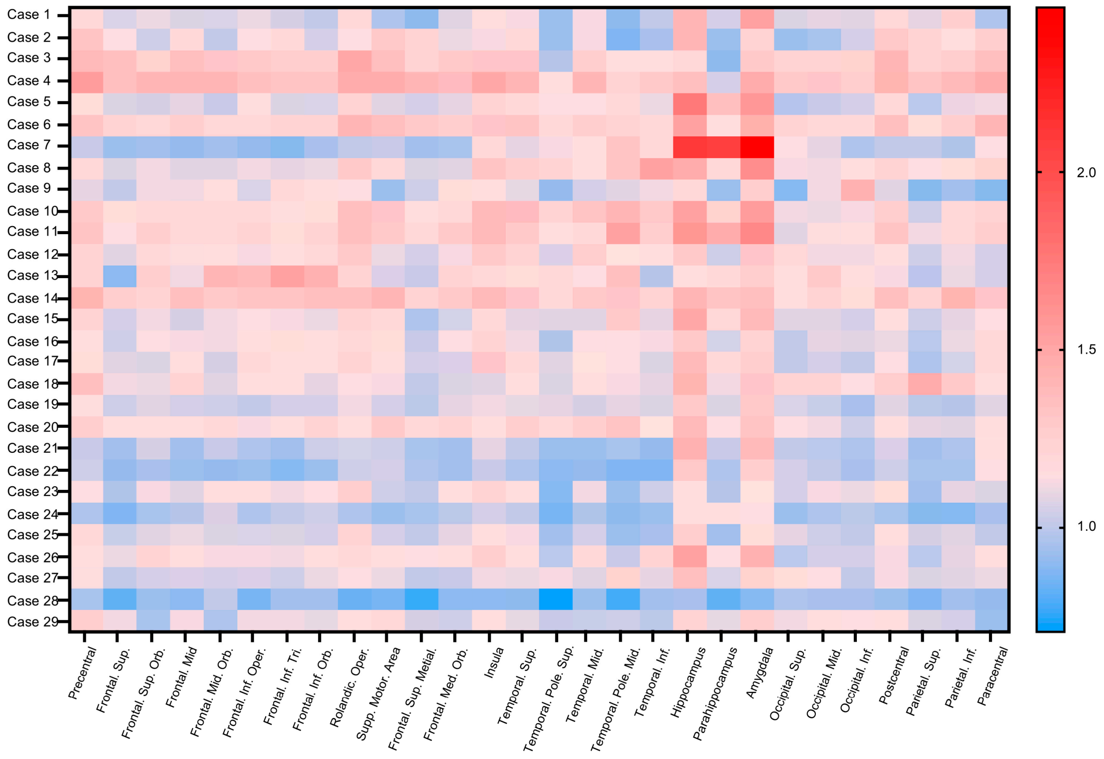

Figure S2. Heat map of SUVRc of [^{18}F]DPA-714 uptake among patients with TLE. It indicated an increased uptake of [^{18}F]DPA-714 in the dominant side of the brain, where the epileptic focus was presumed to be located based on patients' symptoms and VEEG findings. SUVRc, cerebellum normalized mean standardized uptake values ratio; TLE, temporal lobe epilepsy

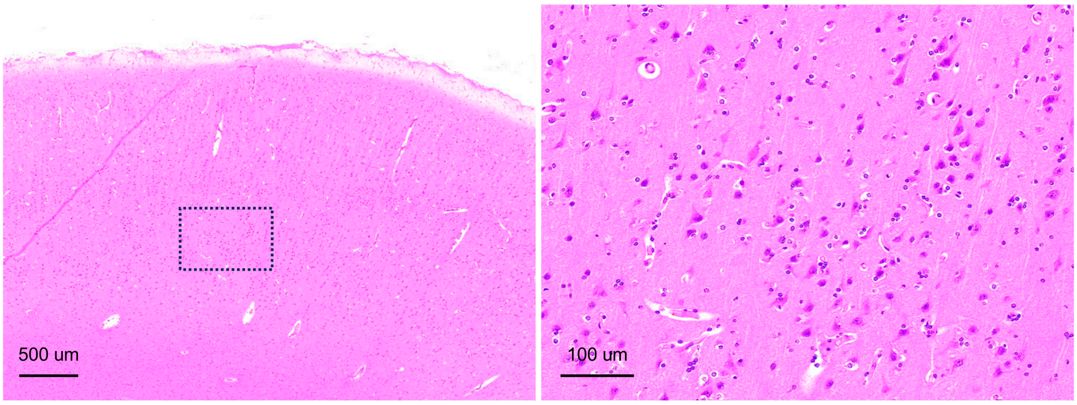

Figure S3. Pathological evaluation of surgical resection tissues using Hematoxylin-eosin staining. It revealed that the morphology of neurons in the lesional cortex was largely normal, with no obvious dysmorphic neurons or balloon cells observed.

Table S1. Demographic, clinical features, VEEG monitoring, and imaging characteristics of TLE patients.

Patients	Age at onset (y)/sex	Seizure types	Seizure frequency during last 3 months	Duration from onset to PET scan (y)	Duration from last seizure to PET scan (d)	Number of antiseizure medications	VEEG monitoring	FDG-PET (hypometabolism)	TSPO-PET (visual read)	Radioligand binding affinity
Case 1	17/F	FBTCS	Daily	0.25	2	2	Left frontotemporal sharp and slow waves complex	Left temporal and parietal lobe	Left temporal and parietal lobe	HAB
Case 2	37/F	FBTCS	Daily	0.5	10	2	Left temporal spikes and sharp waves	Normal	Left temporal lobe	HAB
Case 3	47/M	FIAS, FBTCS	Daily	8	5	2	Left frontotemporal spikes and left temporal slow waves	Left temporal	Left temporal, parietal, and occipital lobe	HAB
Case 4	36/M	FIAS	Monthly	10	14	2	Normal	Left hippocampus	Left occipital lobe	MAB
Case 5	24/F	FIAS	Daily	5	7	3	Left temporal sharp and slow waves complex	Left temporal lobe	Left temporal lobe	HAB
Case 6	36/F	FIAS	Monthly	1	5	1	Left temporal sharp waves, and sharp and slow wave complex	Left temporal lobe	Left temporal lobe	HAB
Case 7	2/M	FIAS, FBTCS	Daily	10	3	2	Left anterior brain and temporal theta rhythm, transforming into slow waves	Left temporal lobe, thalamus	Left temporal lobe	HAB
Case 8	6/M	FIAS, FBTCS	Weekly	9	14	1	Left frontotemporal sharp waves	Left temporal lobe, diffuse	Left temporal lobe	HAB
Case 9	12/M	FIAS, FBTCS	Daily	10	7	3	Left temporoparietal and occipital sharp waves	Normal	Left occipital lobe	HAB
Case 10	27/F	FIAS	Monthly	1	5	2	Left central, frontotemporal sharp waves	Left posterior temporal lobe	Left temporal and occipital lobe	MAB

Case 11	24/F	FIAS	Weekly	20	60	1	Left hemisphere sharp waves	Left temporal lobe	Left temporal lobe	HAB
Case 12	27/M	FIAS	Weekly	10	9	2	Normal	Left temporal and insular lobe	Left temporal and parietal lobe	HAB
Case 13	36/M	FAS, FBTCS	Weekly	1	8	3	Left central, frontal sharp waves	Left frontal and temporal lobe, thalamus, caudatum and putamen	Left frontal lobe	HAB
Case 14	15/M	FIAS	Weekly	17	6	1	Right frontal, central and anterior medial temporal spikes, sharp waves, and sharp and slow waves complex	Right temporal lobe, diffuse	Right temporal lobe	MAB
Case 15	8/F	FIAS, FBTCS	Monthly	17	21	2	Right frontotemporal spikes and sharp waves	Right temporal lobe, diffuse	Right temporal lobe	HAB
Case 16	39/F	FIAS	Weekly	6	7	2	Right anterior medial temporal sharp waves, and sharp and slow wave complex	Right temporal lobe	Right temporal lobe	HAB
Case 17	35/F	FIAS	Weekly	3	6	2	Right temporal spikes and sharp waves	Right frontal and temporal lobe	Right temporal lobe	HAB
Case 18	20/M	FBTCS	Weekly	1	5	3	Widespread slow wave in right hemisphere, significant in temporal lobe	Normal	Right parietal lobe	HAB
Case 19	14/M	FIAS, FBTCS	Weekly	6	7	2	Right frontotemporal spikes and sharp waves	Right temporal lobe, diffuse	Right temporal lobe	HAB
Case 20	24/M	FIAS, FBTCS	Daily	3	1	2	Right temporal sharp waves	Right temporal lobe	Right temporal lobe	HAB
Case 21	12/F	FIAS	Yearly	1	150	2	Left temporal spikes, spike and slow waves complex	Normal	Normal	HAB

Case 22	13/F	FIAS FBTCS	Monthly	0.67	15	3	Left frontal and central spike and slow waves complex	Left temporal lobe, mild	Normal	HAB
Case 23	25/M	FBTCS	Monthly	9	30	2	Left anterior temporal spikes and slow waves complex (phase reversal at F7)	Left temporal lobe, mild	Normal	HAB
Case 24	42/F	FBTCS	Weekly	7	14	2	Right temporal sharp waves	Bilateral temporal lobes, significant in right	Normal	MAB
Case 25	23/M	FIAS	Yearly	9	3	3	Left frontotemporal spikes, and slow waves complex	Normal	Normal	HAB
Case 26	35/F	FIAS FBTCS	Weekly	8	20	2	Left temporal spikes, and slow waves complex	Left mesial temporal lobe, mild	Normal	HAB
Case 27	16/F	FIAS	Weekly	7	12	3	Bilateral temporal sharp waves, sharp and slow waves complex	Left temporal lobe	Normal	MAB
Case 28	30/M	FIAS, FBTCS	Monthly	7	10	1	Right temporal spikes, spike, and slow waves complex	Right frontal lobe, mild	Normal	HAB
Case 29	11/F	FIAS	Yearly	3	14	2	Right parieto-occipital and posterior temporal sharp waves, and spike and slow wave complex	Right occipital lobe, mild	Normal	HAB

TLE, temporal lobe epilepsy; VEEG, video-electroencephalography; TSPO-PET, translocator protein 18 kDa-PET; FBTCS, focal to bilateral tonic-clonic seizure; FIAS, focal impaired awareness seizure; HAB, high-affinity binder; MAB, medium-affinity binder

Table S2. Comparison of TSPO-PET/MRI asymmetry index between TLE patients and healthy controls.

Patients	Max asymmetry index	Brain region with Max asymmetry index	Control mean	95% CI
Case 1	0.208	Amygdala	0.045	0.011 to 0.079
Case 2	0.191	Amygdala	0.045	0.011 to 0.079
Case 3	0.200	Amygdala	0.045	0.011 to 0.079
Case 4	0.216	Occipital_Inf	0.001	-0.047 to 0.048
Case 5	0.314	Hippocampus	0.033	-0.008 to 0.075
Case 6	0.180	Amygdala	0.045	0.011 to 0.079
Case 7	0.692	Parahippocampus	-0.046	-0.080 to -0.013
Case 8	0.378	Amygdala	0.045	0.011 to 0.079
Case 9	0.366	Occipital_Inf	0.001	-0.047 to 0.048
Case 10	0.337	Temporal_Pole_Mid	-0.009	-0.052 to 0.034
Case 11	0.357	Temporal_Pole_Mid	-0.009	-0.052 to 0.034
Case 12	0.152	Temporal_Pole_Mid	-0.009	-0.052 to 0.034
Case 13	0.376	Frontal_Inf	0.006	-0.054 to 0.065
Case 14	0.212	Temporal_Pole_Mid	-0.009	-0.052 to 0.034
Case 15	0.235	Temporal_Pole_Mid	-0.009	-0.052 to 0.034
Case 16	0.256	Temporal_Pole_Mid	-0.009	-0.052 to 0.034
Case 17	0.275	Temporal_Pole_Mid	-0.009	-0.052 to 0.034
Case 18	0.151	Parietal_Sup	0.052	0.003 to 0.101
Case 19	0.206	Temporal_Pole_Mid	-0.009	-0.052 to 0.034
Case 20	0.151	Temporal_Mid	-0.001	-0.028 to 0.026
Case 21	0.082	Amygdala	0.045	0.011 to 0.079
Case 22	0.064	Frontal_Sup_Orb	-0.016	-0.040 to 0.008
Case 23	0.078	Frontal_Sup_Orb	-0.016	-0.040 to 0.008
Case 24	0.127	Parietal_Sup	0.052	0.003 to 0.101
Case 25	0.069	Amygdala	0.045	0.011 to 0.079
Case 26	0.128	Temporal_Inf	-0.001	-0.010 to 0.008
Case 27	0.132	Paracentral_Lobule	0.119	0.080 to 0.157
Case 28	0.102	Frontal_Mid_Orb	0.001	-0.031 to 0.034
Case 29	0.140	Occipital_Mid	0.024	-0.007 to 0.056

TSPO: translocator protein 18 kDa; TLE, temporal lobe epilepsy; Inf, inferior; Sup, superior; Mid, middle; Orb, orbital.
